# Function analysis of differentially expressed microRNAs in TGF-β1-induced cardiac fibroblasts differentiation

**DOI:** 10.1042/BSR20182048

**Published:** 2019-10-11

**Authors:** Suxuan Liu, Wen Ke, Yang Liu, Zhenzhen Zhao, Lina An, Xiaohua You, Fan Yang, Xiangqun Yang, Guokun Wang, Xianxian Zhao

**Affiliations:** 1 Department of Cardiology, Changhai Hospital, Naval Medical University, Shanghai 200433, China; 2 Department of Medical Service, 905th Military Hospital, Naval Medical University, Shanghai 200040, China; 3 Department of Cardiovascular Surgery, Institute of Cardiac Surgery, Changhai Hospital, Naval Medical University, Shanghai 200433, China; 4 Anesthesiology and Intensive Care Medicine, Changhai Hospital, Naval Medical University, Shanghai 200433, China; 5 Department of Geriatrics, Shanghai First People’s Hospital, Medical College, Shanghai Jiaotong University, Shanghai 200080, China; 6 Department of Anatomy, Naval Medical University, Shanghai 200433, China

**Keywords:** cardiac fibroblasts, deep sequencing, differentiation, microRNAs, transforming growth factor-β1

## Abstract

**Background:** Cardiac fibroblasts differentiation plays a critical role in cardiac remodeling and failure, but the underlying molecular mechanisms are still poorly understood. MicroRNAs (miRNAs) had been identified as important regulators during cell differentiation. The aim of the present study was to screen the miRNAs involved in regulation of cardiac fibroblasts differentiation. **Methods:** The differentiation of rat cardiac fibroblasts into myofibroblasts was induced by transforming growth factor-β1 (TGF-β1). Small RNA sequencing was then applied to detect the differentially expressed miRNAs. **Results:** A total of 450 known miRNAs were detected, and 127 putative novel miRNAs were predicted by miRDeep2 analysis. DEGseq analysis and qRT-PCR confirmed that 24 known miRNAs were differentially expressed in TGF-β1-induced cardiac fibroblasts, including three up-regulated miRNAs and 21 down-regulated miRNAs. After miRNAs target genes prediction by miRanda algorithm, pathway analysis showed that these potential target genes were involved in Calcium signaling pathway, Type II diabetes mellitus, and Glutamatergic synapse pathway, etc. Meanwhile, seven putative miRNAs were also detected differentially expressed during TGF-β1-induced cardiac fibroblasts differentiation. **Conclusions:** These differentially expressed miRNAs might play critical roles in cardiac fibroblasts differentiation. Altered expression of miRNAs may yield new insights into the underlying mechanisms of cardiac fibrosis and provide novel mechanism-based therapeutic strategies for cardiac fibrosis.

## Introduction

Cardiovascular diseases have become a major cause of morbidity and mortality in the world. Cardiac fibrosis is defined as excessive deposition of fibrous connective tissue and represents a fundamental constituent in many cardiac pathophysiologic conditions, such as cardiomyopathies, heart failure, and myocardial infarction [1,2]. Cardiac fibrosis following acute myocardial infarction provides myocardial healing in the short term and prevents from ventricular wall rupture [3]. Cardiac fibrosis in long-standing heart failure accumulates throughout the heart and leads to myocardium stiffening and progressively worsens cardiac function [4]. The main determinant of cardiac fibrosis is the differentiation of cardiac fibroblasts into myofibroblasts, characterized by excessive fibroblasts proliferation, extracellular matrix (ECM) deposition and contraction due to the expression of α-smooth muscle actin (α-SMA) [5,6]. Numerous studies suggested many cytokines, including transforming growth factor-β1 (TGF-β1), connective tissue growth factor (CTGF), and platelet-derived growth factor (PDGF), actively participate in the transformation of quiescent fibroblasts to myofibroblasts and cardiac fibrosis [7,8]. Mechanical stimuli have also been shown to activate the cardiac fibroblasts differentiation [9]. Although cardiac myofibroblasts are known to contribute to many pathological processes, the underlying mechanisms of cardiac fibroblasts differentiation are still poorly understood. Numerous studies suggested that cardiac fibrosis can be prevented by inhibition of TGF-β [10,11]. However, TGF-β is involved in many biological processes such as embryogenesis, angiogenesis, and immune modulation. Long-term inhibition of TGF-β1 and its receptors can lead to some unacceptable adverse effects [12]. In this regard, the downstream effectors of TGF-β1-induced cardiac fibroblasts differentiation have emerged as important targets for antifibrotic therapies.

MicroRNAs (miRNAs) are a class of small non-coding RNAs (∼22 nucleotides in length) that regulate gene expression post-transcriptionally via impeding translation [13]. Increasing evidence suggested that dysregulation of miRNAs was associated with the pathophysiological process of cardiovascular diseases, such as coronary heart disease, arteriosclerosis, and ischemia-reperfusion injury [14,15]. Recently, aberrant miRNAs were identified as important regulators of cardiac fibroblasts differentiation and cardiac fibrosis. It has been reported that *miR-433, miR-21* and *miR-125b* could promote cardiac fibrosis [16–18], while *miR-150, miR-29a* and let-7i could suppress the fibrotic response of heart [19–21]. These studies indicate that miRNAs are powerful regulators of cardiac fibrosis. However, the overall profiles of differentially expressed miRNAs during TGF-β1-induced cardiac fibroblasts differentiation have not been investigated. In the present study, we aimed to identify miRNAs expression profiles during TGF-β1-induced cardiac fibroblasts differentiation by small RNA deep sequencing, and further gain more insight into the miRNA biological functions and therapeutic potentials for cardiac fibrosis by bioinformatics tools.

## Materials and methods

### Isolation of rat cardiac fibroblasts

The experiment was conducted at Institute of Cardiac Surgery in Changhai Hospital according to NIH Guidelines for Care and Use of Laboratory Animals and was approved by the Institutional Animal Ethical Committee of Second Military Medical University (SMMU_2016004). Cardiac fibroblasts were isolated from neonatal Sprague–Dawley rats and cultured as described previously [22]. In brief, neonatal hearts were rapidly removed, minced and digested with collagenase at 37°C. Cells were separated by gradient centrifugation and selective attachment procedures. Cardiac fibroblasts were resuspended in Dulbecco’s Modified Eagle’s Medium (DMEM) supplemented with antibiotic (penicillin and streptomycin) and 10% fetal bovine serum. Cells were maintained in a humidified atmosphere of 5% CO_2_ at 37°C. Fibroblasts were used at 3–5 passages in the further experiments. Based on published study, cultured cardiac fibroblasts were serum-deprived for 24 h and then stimulated with TGF-β1 at 10 ng/ml for 48 h [23]. The control cells were cultured in the medium without TGF-β1.

### Immunofluorescence staining

After treatment of TGF-β1 for 48 h, the phenotype of cardiac myofibroblasts was characterized with immunofluorescence staining of α-SMA. The culture medium was removed and the cells were fixed by paraformaldehyde for 10 min, followed by incubation with PBS containing 0.3% Triton X-100 at room temperature for 20 min. Then cells were incubated with rabbit anti-rat α-SMA antibody (1:500) at 4°C overnight, followed by Alexa Fluor 488 conjugated IgG (1:1000) at 37°C for 1 h. Nuclei were stained by DAPI (5 μg/ml) and then visualized with a fluorescence microscope.

### Small RNA deep sequencing

Total RNA was isolated from cardiac fibroblasts by miRNeasy Kit (Qiagen, Germany) according to the manufacturer’s instructions. About 10 μg of total RNA were ligated with proprietary adapters, reverse transcribed to cDNA and amplified by PCR. Subsequently, the PCR products were purified by RNA gel electrophoresis and validated for library construction. Finally, the libraries were deep sequenced using HiSeq 4000 (Illumina, U.S.A.) at Shanghai OE Biotech Co., Ltd. MultiExperiment Viewer software was applied for comparison of miRNA expression values, preparation of heat-map and hierarchical clustering analyses (fold change > 1.5 or < 0.66; *P-*value < 0.05; q-value < 0.01).

### Quantitative real-time PCR (qRT-PCR)

To validate the deep sequencing results in the study, qRT-PCR was performed on a LightCycler 480 II PCR system (Roche, Basel, Switzerland) by using SYBR Green (TAKARA, Japan). Complementary total RNA was used to generate cDNA by using PrimeScript RT reagent Kit (TAKARA, Japan) with special stem-loop primer for miRNA and oligo-dT or random primer for mRNA. Rnu6b were used as reference genes for miRNAs expression detection. The fold change was calculated by 2^–ΔΔ*C*^t method. Each PCR experiment was repeated for three times.

### Bioinformatics analysis

Small RNAs annotation was identified based on the blast result and miRbase database. After blast with rat genome, the annotated reads which were in alignment with rat miRbase database were identified as ‘known miRNAs’. The unannotated reads which had a stem-loop structure were identified as ‘putative miRNAs’ after sequences homologous analysis and secondary structure prediction by miRDeep2 software. The miRanda algorithm was used to predict potential targets of miRNAs. Gene ontology (GO) functional analysis was performed in the standard enrichment computation method based on the Database for Annotation, Visualization and Integrated Discovery (DAVID). The pathways were enriched according to the miRNAs target genes annotated in the Kyoto Encyclopedia of Genes and Genomes (KEGG, http://www.genome.jp/kegg/) pathway database, and sorted by the *P*-value of hypergeometric distribution.

### Statistical analysis

All statistical analyses were performed by SPSS version 17.0. The differences between any two groups were analyzed via independent *t*-tests. A *P*-values less than 0.05 were considered statistically significant difference.

## Results

### TGF-β1 induced differentiation of cardiac fibroblasts into myofibroblasts

**Figure 1:**
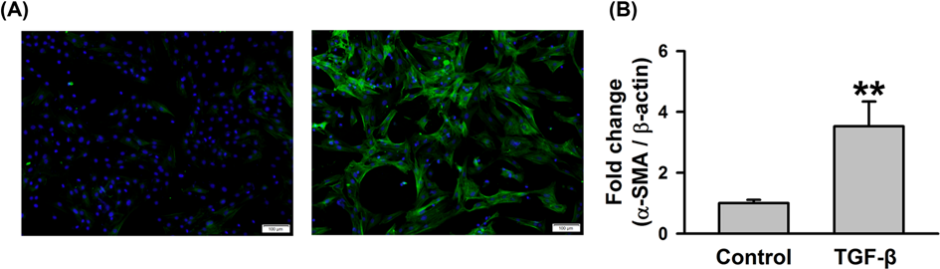
TGF-β1 induced differentiation of cardiac fibroblasts into myofibroblasts (**A**) Representative images of α-SMA immunofluorescence staining during the differentiation of cardiac fibroblasts into myofibroblasts induced by TGF-β1 (10ng/ml). (**B**) The relative expression of α-SMA in cardiac fibroblasts treated by TGF-β1 (10ng/ml) for 48 h. QRT-PCR assay was applied to analyze the expression change of α-SMA (*n*=4 in each group). ^**^P<0.01 vs control group.

As reported previously, TGF-β1 could induce the differentiation of cardiac fibroblasts into myofibroblasts and α-SMA was a typical molecular marker of myofibroblasts. In our study, TGF-β1 induced the differentiation of cardiac fibroblasts into myofibroblasts was confirmed by immunofluorescence staining of α-SMA. Compared with control group, the percentages of cardiac fibroblasts expressing α-SMA were significantly increased in the TGF-β1 group (Figure 1A). We also identified the mRNA levels of α-SMA via qRT-PCR. Compared with control group, the mRNA expressions of α-SMA were up-regulated in the TGF-β1 group (Figure 1B).

### Overview of small RNA sequencing data

**Figure 2:**
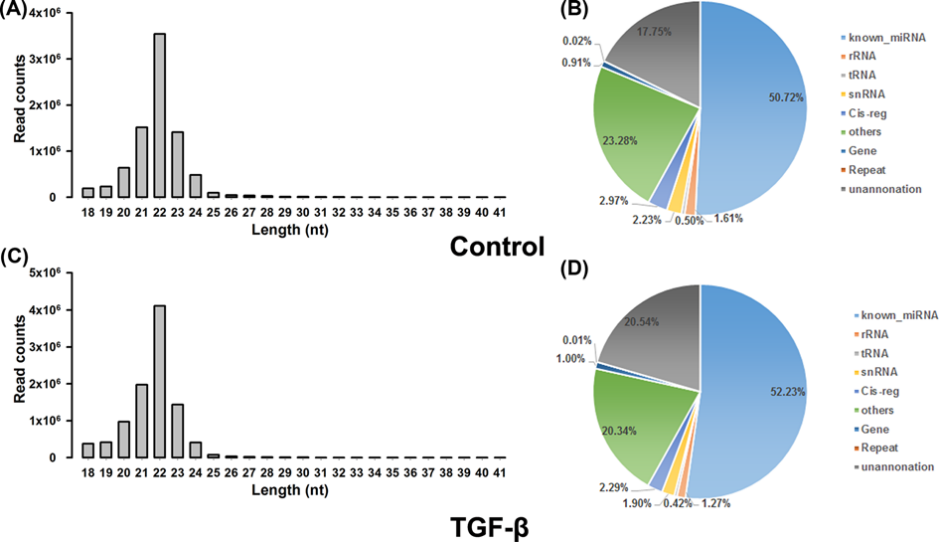
Summary of small RNA deep sequencing data in TGF-β1-induced cardiac fibroblasts **(A,C**) Read length distribution (15–41 nt) and abundance of small RNAs sequences in cardiac fibroblasts with or without TGF-β1 treatment. (**B,D**) Frequency of unique small RNA distribution among the different categories. The unique sequences were subjected to searches for the types and numbers of sRNA using the Rfam databases.

After filtering out low-quality and meaningless reads, about 10 million clean reads (between 18 and 41 nt) were obtained from Illumina Solexa sequencing. Size distribution assessment showed that small RNA sequence length was mainly concentrated at 20–24 nt, and the 22nt-small RNA sequence had the most read counts (Figure 2A,C). Blast results showed that over 80% of reads were in alignment with rat genome. According to their biogenesis and annotation from Rfam databases, the clean sequences were categorized into different groups, including rRNA, miRNA, snRNA, and tRNA, etc. There were 4,186,319 (50.72%) and 5,150,573 (52.23%) reads identified as known miRNAs for the control group and TGF-β1 group, respectively (Figure 2B,D). After deduplication, a total of 367,743 and 411,864 unique reads were obtained for the two groups, and over 70% of these reads had not been annotated (Table 1).

**Table 1:** Summary of data generated from small RNA deep sequencing

Group	Raw reads	Clean reads	Valid reads	Unique reads	Unannonated reads
Control	10,779,121	9,451,230	8,257,745	367,743	261,459
TGF-β1	12,107,760	11,612,196	9,865,935	411,864	305,730

### Characterization of known and putative novel miRNAs

**Figure 3:**
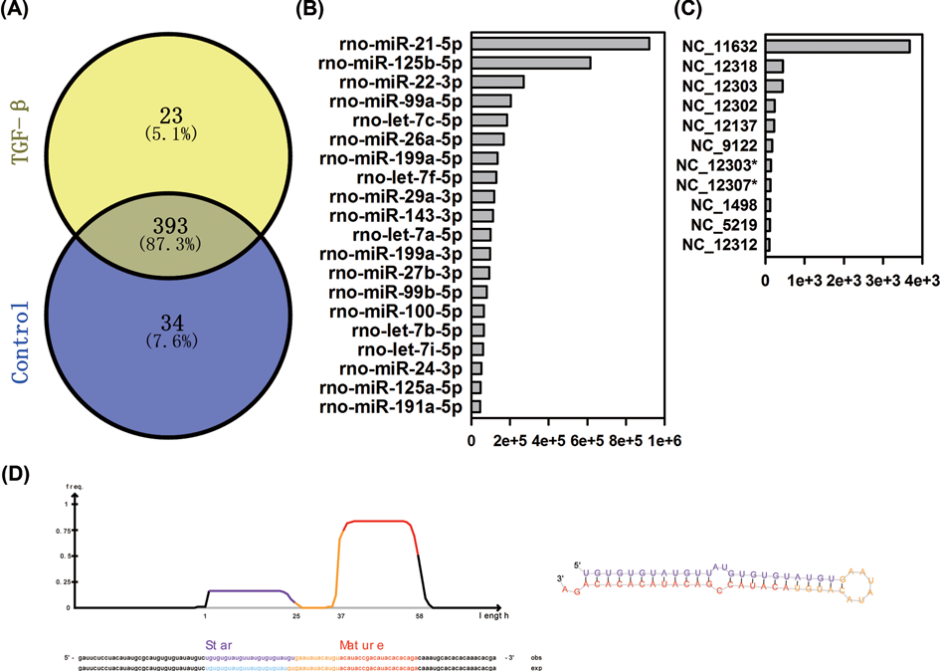
Characterization of known and putative novel miRNAs (**A**) A total of 416 and 427 miRNAs detected in cardiac fibroblasts with or without TGF-β1 stimulation, and 393 miRNAs were expressed in both two groups. (**B**) Description of top 20 abundant known miRNAs in cardiac fibroblasts, and no significant difference in highly expressed miRNA species after TGF-β1 stimulation. *miR-21-5p* was the most abundant expressed miRNAs. (**C**) Description of the putative miRNAs with clean reads above 100. (**D**) The sequence secondary structure of NC_12318 was predicted by randfold software. The precursor had stable stem-loop structure.

According to the database of miRbase 21.0, a total of 450 known miRNAs were identified in the present study. There were 416 and 427 miRNAs detected from neonatal rat cardiac fibroblasts with or without TGF-β1 stimulation, among which 393 miRNAs were expressed in both two groups (Figure 3A). The top 20 abundant miRNAs were illustrated (Figure 3B). The most highly expressed miRNA was *miR-21*. The levels of *miR-125b, miR-22, miR-99a*, and let-7c families were also abundantly expressed. No significant difference was found on highly expressed miRNA species between the two groups. Meanwhile, 127 putative novel miRNAs were predicted from the unannotated reads by miRDeep2 analysis (Supplementary Table S1). The putative miRNAs with clean reads above 100 were also illustrated (Figure 3C). A BLAST (Basic Local Alignment Search Tool) search of rat genome revealed all the putative novel miRNAs shared little homology. Among them, one putative miRNA (NC_12318) with highest miRDeep2 score had four genomic locations on the antisense strand of chromosome 17 (71805292-71805349, 71800308-71800365, 71801732-71801789, and 72062781-72062838). The sequence secondary structure predicted by randfold software showed that the precursor of NC_12318 had stable stem-loop structure (Figure 3D).

### Differentially expressed miRNAs during cardiac fibroblasts differentiation

**Figure 4:**
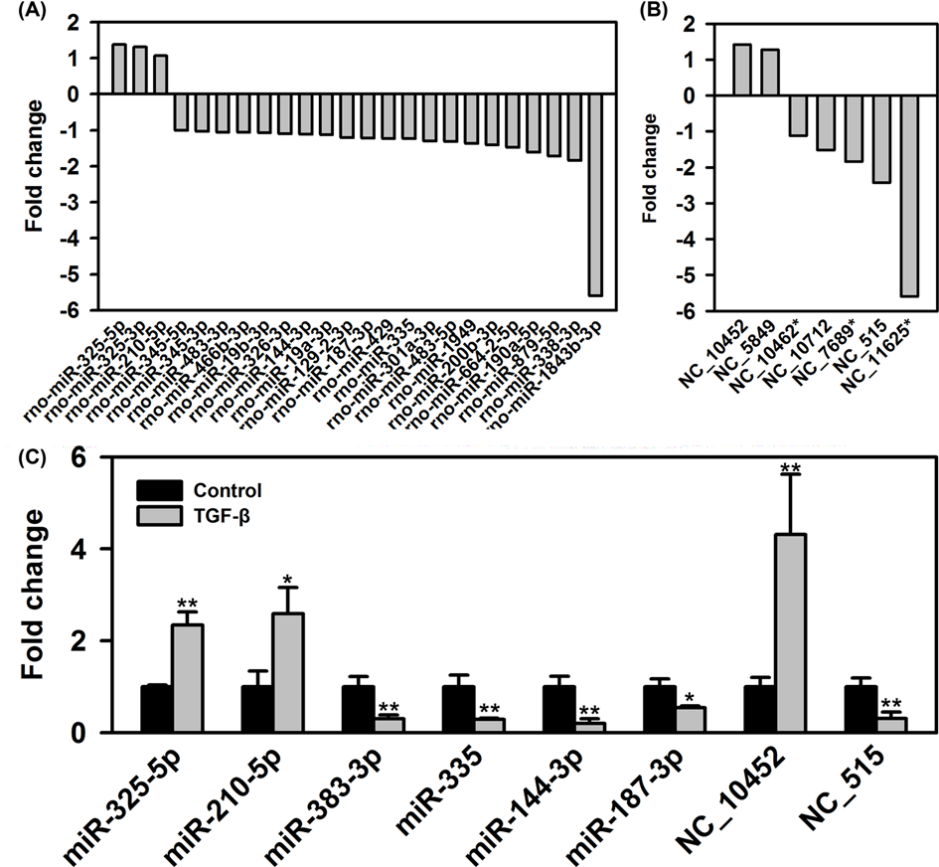
Differentially expressed miRNAs during cardiac fibroblasts differentiation (**A,B**) A total of 24 known miRNAs and 7 putative miRNAs were differentially expressed during TGF-β1-induced cardiac fibroblasts differentiation. The data were obtained from small RNA sequencing and transformed by log2 [fold change (TGF-β/Control)]. The positive number in Y-axis represented up-regulation, while the negative number represented down-regulation. (**C**) Validation of differentially expressed miRNAs by qRT-PCR (*n*=4 in each group). ^*^P<0.05, ^**^P<0.01 vs control group.

DEGseq analysis results showed that a total of 24 known miRNAs were differentially expressed during TGF-β1 induced cardiac fibroblasts differentiation, including three up-regulated miRNAs and 21 down-regulated miRNAs. The details of differentially expressed miRNAs are shown in Figure 4A. Meanwhile, seven putative miRNAs were found to be differentially expressed during TGF-β1-induced cardiac fibroblasts differentiation, including two up-regulated miRNAs and five down-regulated miRNAs (Figure 4B). Among these differentially expressed miRNAs, eight miRNAs (six known miRNAs and two putative miRNAs) were randomly selected for validation via qRT-PCR. All these miRNAs showed a consistent expression pattern with the results from small RNA sequencing (Figure 4C), indicating high reliability of our analysis.

### Prediction and annotation of miRNA target genes

**Figure 5:**
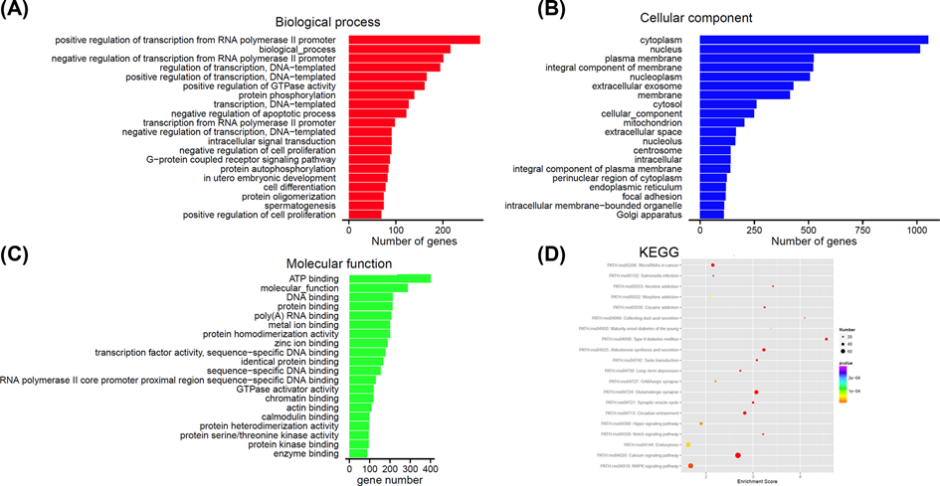
Function annotation of the potential target genes of miRNAs A total of 241 target genes were identified for the up-regulated miRNAs and 2542 target genes identified for the down-regulated miRNAs. (**A**–**C**) GO analysis was performed based on the DAVID. (**D**) Pathway analysis was performed based on KEGG databases, and sorted by the *P*-value of hypergeometric distribution. The bubble chart was made by ggplot2 software on the top 20 enriched pathways. The enrichment score is calculated according to the formula ‘Enrichment score = (m/n)/(M/N)’. (N, the number of genes with annotation in KEGG; n, the number of potential target genes with annotation in KEGG; M, the number of genes annotated as a designated KEGG Term; m, the number of potential target genes annotated as a designated KEGG Term).

To better analyze the functions of miRNAs, potential target genes were predicted by the miRanda software. A total of 241 target genes were identified for the up-regulated miRNAs and 2542 target genes identified for the down-regulated miRNAs. We found the most enriched GO was correlated with transcription regulation in the biological process analysis. The majority of genes were proved to be related to the cytoplasm region in the cellular component analysis and ATP binding in the molecular function (Figure 5A–C). The biological functions of these target genes were further investigated using KEGG pathway analysis. A total of 257 pathways were significantly enriched, and most enriched in Calcium signaling pathway, Type II diabetes mellitus, and Glutamatergic synapse pathway, etc. (Figure 5D).

## Discussion

As a profibrotic cytokine, TGF-β1 could induce differentiation of cardiac fibroblasts into myofibroblasts, which plays an important role during the process of cardiac fibrosis. In the present study, the differentially expressed miRNAs were identified in TGF-β1-induced cardiac fibroblasts by small RNA sequencing. The potential targets of these miRNAs were predicted to be related to the cytoplasm region in the cellular component analysis, and ATP binding in the molecular function, and were most enriched in Calcium signaling pathway, Type II diabetes mellitus, and Glutamatergic synapse pathway. The altered expression of miRNAs may yield new insights into the underlying mechanisms of cardiac fibrosis and provide novel mechanism-based therapeutic strategies for cardiac fibrosis.

In the present study, primary cardiac fibroblasts were isolated from neonatal rat hearts using selective attachment procedures. By differential preplating and passaging, the cardiomyocytes could be removed, which was confirmed by immunofluorescence staining of α-actinin. However, other types of cells (mainly endothelial cells) might be also co-isolated with cardiac fibroblasts [24]. Due to miRNA’s differential expression profile in various cells, some miRNAs restricted to endothelial cells would express disorderly in response to TGF-β1 stimulation, which might lead to false positive results in sequencing, such as *miR-126* [25]. Compared with conventional isolation method, the novel technology based on fluorescent-activated cell sorting (FACS) or magnetic beads would gain higher fibroblasts purity. However, the inherent heterogeneity of cardiac fibroblasts limited the application of this technology in some extent. It was reported that isolation of fibroblasts by FACS and magnetic beading with Thy-1 antibody could yield greater than 99% purity [26]. However, effective surface markers were scarcely found for FACS-based isolation of cardiac fibroblasts until now. The negative-gate selection strategy might be a viable method to remove the co-isolated endothelial cells.

It is widely reported that TGF-β1 could control some cardio-pathologic and cardio-physiologic miRNAs at different steps and affect different components in cardiovascular system [27–30]. So far, the mechanisms of the above process have not been clearly elucidated. MiRNAs, as important gene expression regulators, would also be dysregulated in response to internal and external stimuli. In the present study, a total of 24 known miRNAs and 7 putative miRNAs were found differentially expressed in cardiac fibroblasts in response to TGF-β1 stimulation. Some known miRNAs have been confirmed to participate in the regulation of fibrosis, such as *miR-210, miR-335*, and *miR-429*, etc. Some other miRNAs have been found to regulate TGF-β signaling pathway, such as *miR-144* [31], *miR-338*, and *miR-190*, etc. The putative miRNAs might also be important regulators during cardiac fibroblasts differentiation, but should be verified on their biogenesis, followed by function exploration.

Gain-of-function and loss-of-function experiments were most commonly used for genes function and mechanism investigation. Liposomes-mediated transfection of antagonist and antagomir was an important strategy for *in vitro* miRNA expression intervention, but less applied for primary cultured cells. Recombinant adenovirus- or lentivirus-mediated miRNAs overexpression and inhibition could obtain better intervention effect in primary cultured cells. Recently, adenovirus associated virus (AAV) has been widely used in miRNAs expression modification *in vivo* [32]. AAV serotype type 2/9 could effectively influence miRNAs expression levels in heart. In the present study, the differentially expressed miRNAs were identified during cardiac fibroblasts differentiation. It was very valuable to validate the role of these miRNAs on cardiac fibroblasts phenotype change. Therefore, the further studies should be focused on verification of miRNAs function in future.

Identification of miRNA-mediated regulation networks is based on their target genes analysis. As commonly accepted, miRNAs could inhibit target genes expression through binding to their 3′-untranslated regions (3′UTR). According to the incomplete complementary characteristic between miRNAs and target genes, many bioinformatics algorithms have been developed for miRNAs target genes prediction, such as miRanda, targetscans, etc. Meanwhile, biological function and pathway analysis of the potential target genes of unknown function miRNA would also provide direction for further research. Recently, long non-coding RNAs (lncRNAs) and circRNAs have been reported to function in gene expression regulation as competing endogenous RNAs (ceRNAs) of miRNAs [33]. Benefitting from the conservation of miRNA seed sequences, the potential interaction between miRNAs and lncRNAs (or circRNAs) could be predicted by bioinformatics analysis. These bioinformatics tools would provide valuable information for further investigation on miRNAs roles and mechanisms during cardiac fibroblasts differentiation.

In summary, miRNA expression and function could be reprogramed and used as therapeutic targets for cardiac fibrosis. Pharmacological modulation of specific miRNA activity might have potential clinical relevance. Future experiments will be performed to investigate the precise mechanisms of the dysregulated miRNAs in cardiac fibroblasts.

## Supplementary Material

Supplementary Table S1

## Abbreviations

α-SMA: α-smooth muscle actin
AAV: adenovirus associated virus
cDNA: complementary DNA
circRNA: circular RNA
DAPI: 4′,6-Diamidino-2-Phenylindole, Dihydrochloride
DAVID: Database for Annotation, Visualization and Integrated Discovery
FACS: fluorescent-activated cell sorting
GO: gene ontology
KEGG: Kyoto Encyclopedia of Genes and Genomes
LncRNA: long non-coding RNA
miRNA: microRNA
qRT-PCR: quantitative real-time Polymerase Chain Reaction
rRNA: ribosomal RNA
snRNA: small nuclear RNA
TGF-β1: transforming growth factor-β1
tRNA: transfer RNA
